# From F. S. C., Edwards, Mississippi

**Published:** 1882-06

**Authors:** 


### CORRESPONDENCE.

EDWARDS, Miss., May 8, 1882.

EDITOR DENTAL REGISTER.—*Dear Sir:*—I have a case in my practice which is very peculiar. As I have never seen a similar case, I have concluded to give you a description of it, that the many readers of your valuable journal may give their opinion of the same.

The patient is a lady of light complexion, aged 62 years, and not in the best of health. I was called to see her about one year ago, and extracted the left lower wisdom tooth, which I found quite loose, and free from decay, although it was giving considerable pain to the patient. Since that time she has had several extracted by another dentist. A few days ago I was again called to see her, and this time extracted the last left upper molar, and first right lower molar, as in the first case, I found these teeth a little loose but free from decay, there is yet remaining in the mouth sixteen teeth nine in upper maxillary and seven in the lower. All these teeth are sound, the gums around each present a very healthy appearance, but they are somewhat loose, the upper maxillary posterior from the last remaining teeth is about twice the thickness it should be in health. And the lower maxillary has protuberances on each lingual or inner side four in number, two on each side, the largest being the posterior which is about half the size of a bird's egg, the smallest about half that size, these protuberances are very hard and will not yield to the touch of an instrument. The patient informed me that she was salivated about thirty years ago and has suffered from neuralgic pains for a long time periodically, and wishes to know if she will get relief by having her teeth all extracted, and if she can wear a plate on the lower maxillary after the teeth are out. I cannot locate the cause in any of the teeth, as she points to another troublesome tooth, as fast as they are extracted. Having given the particulars as near as I can, I would like to hear from some one who has had a more extended range of experience.

Respectfully, F. S. C.

